# Concurrent urinary and bowel diversion: Surgical modification with sigmoid colon that avoids a bowel anastomosis

**DOI:** 10.1590/S1677-5538.IBJU.2019.0194

**Published:** 2020-01-13

**Authors:** Didi P. Theva, Angela Kuhnen, Richard K. Babayan, Mark H. Katz

**Affiliations:** 1 Department of Urology, Boston Medical Center/Boston University School of Medicine Boston; 2 Department of Surgery, Division of Colorectal Surgery, Boston Medical Center/Boston University School of Medicine Boston

**Keywords:** Urinary Diversion, Intestines, Anastomosis, Surgical, Urinary Tract

## Abstract

**Objective:**

Cystectomy with urinary diversion is the gold standard for muscle invasive bladder cancer. It also may be performed as part of pelvic exenteration for non-urologic malignancy, neurogenic bladder dysfunction, and chronic conditions that result in a non-functional bladder (e.g., interstitial cystitis, radiation cystitis). Our objective is to describe the surgical technique of urinary diversion using large intestine as a conduit whilst creating an end colostomy, thereby avoiding a primary bowel anastomosis and to show its applicability with respect to urologic conditions.

**Materials and Methods:**

We retrospectively reviewed five cases from a single institution that utilized the described method of urinary diversion with large intestine. We describe operative times, hospital length of stay (LOS), and describe post-operative complications.

**Results:**

Five patients with a variety of urologic and oncologic pathology underwent the described procedures. Their operative times ranged from 5 hours to 11 hours and one patient experienced a Clavien III complication.

**Conclusion:**

We describe five patients who underwent this procedure for various medical indications, and describe their outcomes, and believe dual diversion of urinary and gastrointestinal systems with colon as a urinary conduit to be an excellent surgical option for the appropriate surgical candidate.

## INTRODUCTION

Cystectomy with urinary diversion is the gold standard for muscle invasive bladder cancer. It also may be performed as part of pelvic exenteration for non-urologic malignancy, neurogenic bladder dysfunction, and chronic conditions that result in a non-functional bladder (e.g., interstitial cystitis, radiation cystitis). The hallmark of all forms of urinary diversion is the use of bowel to substitute for the excised bladder. Potential gastrointestinal segments include stomach, jejunum, ileum, and colon. The choice of segment is based on multiple patient factors, including the type of diversion, prior abdominal and bowel surgery, and potential risk of metabolic abnormalities post-operatively. Ileal conduit is the most common form of urinary diversion, first described by Seiffert in 1935 and then popularized by Bricker. As stated by Hautmann (1), the most important factors when considering conduits are adequate cancer control, lower complication rates and feasibility to continue activities of daily living comfortably.

Urinary diversions have evolved significantly over time. Early diversions of the late 1800's and early 1900's typically anastomosed a portion of the urinary tract to a segment of bowel that remained in continuity with the remainder of the GI tract. Examples include ureteroproctostomy for bladder extrophy, direct anastomosis of the trigone with bowel using two layers (Maydl) and anastomosis of the ureters to cecum (2).

Historically, rectal and rectosigmoid bladder conduits were introduced in the 1890's but with minimal success until the 1950s when Boyce and Vest described and performed a two-stage technique for a rectal bladder in exstrophy patients (3). Gastric and transverse colon conduits became popular in the 1960s when radiotherapy was used commonly for pelvic malignancies, as these were less affected by radiation field.

In 1940, Bricker used the isolated sigmoid colon as a conduit in four patients and placed the urostomy stoma near the colostomy (4). This technique was then revised by Turner-Warwick in the 1950s when he placed the sigmoid colostomy on the left abdomen and the urinary conduit with sigmoid colon in the right abdomen, as we will describe below (5). As time passed, others explored continent diversions using ileum and ileocecal neobladders. The late 20^th^ century ushered in the modern-day era of continent urinary diversion, both continent cutaneous and orthotopic neobladder.

Through history, the ileal segment has become the most utilized for multiple reasons including ease of mobilization and less risk of intestinal malabsorption, electrolyte abnormalities and chronic diarrhea. Despite this, there are several literature reviews highlighting the negative early postoperative and long-term effects of primary bowel anastomosis which is typically performed after isolating a bowel segment for urinary diversion, including postoperative ileus, increased hospital length of stay, fistula formation, anastomotic breakdown, bowel obstruction, intestinal stenosis (6).

In cases in which the patient has a primary bowel condition in addition to one of the urinary indications for diversion, it may be beneficial to consider using sigmoid colon as previously described, to create two concurrent diversions-urostomy and colostomy. Patients requiring urinary diversion may have concomitant bowel conditions that favor diversion such as malignancy, neurogenic bowel with severe constipation, non-healing wounds, fistulas and chronic diarrhea. Considering these indications, there are several patient conditions that may benefit from simultaneous urinary and bowel diversion such as neurologic diseases (e.g., spinal cord injury, multiple sclerosis), which result in neurogenic bladder and bowel, and locally advanced pelvic malignancy involving and/ or invading the genitourinary and gastrointestinal tract. Barboglio Romo et al. reviewed 46 cases of patients with double diversions, comparing those who underwent urinary diversion after colostomy versus simultaneous creation, and found no independently associated risk of adverse events with concurrent creation (7).

Here we describe the surgical technique for an infrequently utilized technique for simultaneous urinary and bowel diversion with a colonic conduit to avoid a bowel anastomosis and minimize the amount of bowel taken out of continuity. Theoretical benefits include decreased complications and faster recovery. We describe our operative technique in detail, review five cases performed at our institution, and discuss the potential advantages of this surgical procedure.

## Surgical Technique

The patient is given a mechanical bowel preparation with either magnesium citrate or polyethylene glycol. An orogastric tube is placed by anesthesia. A urethral catheter is placed to gravity drainage after sterile preparation and draping. The table is slightly flexed. A lower midline incision is utilized from the umbilicus to the pubic symphysis. The peritoneum is carefully entered and a Bookwalter retractor is placed. Adhesions are lysed as necessary. The ureters dissected free in the retroperitoneum and divided distally at the ureterovesical junction. A complete or supra-trigonal cystectomy is performed depending on the indication for surgery. At this point, our general or colorectal surgery colleagues join the operation.

**Figure 1:**
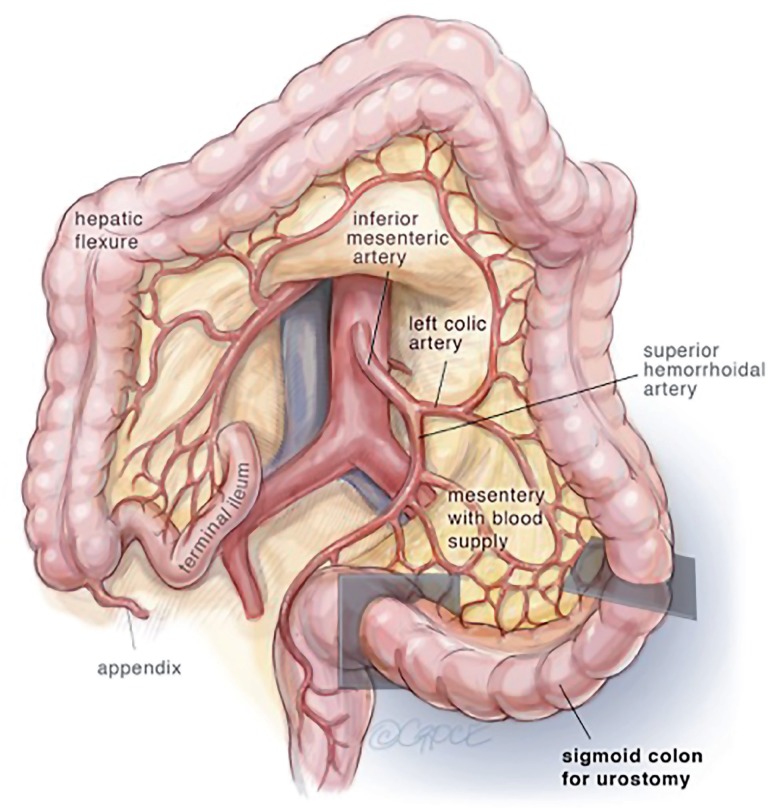
Large bowel with blood supply.

**Figure 2:**
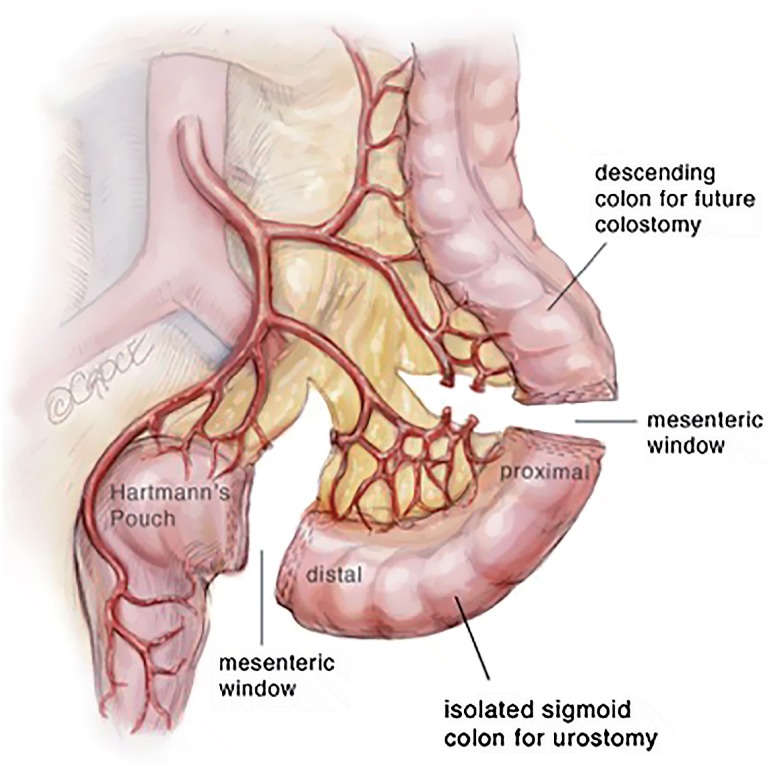
Sigmoid isolation for urinary conduit.

**Figure 3:**
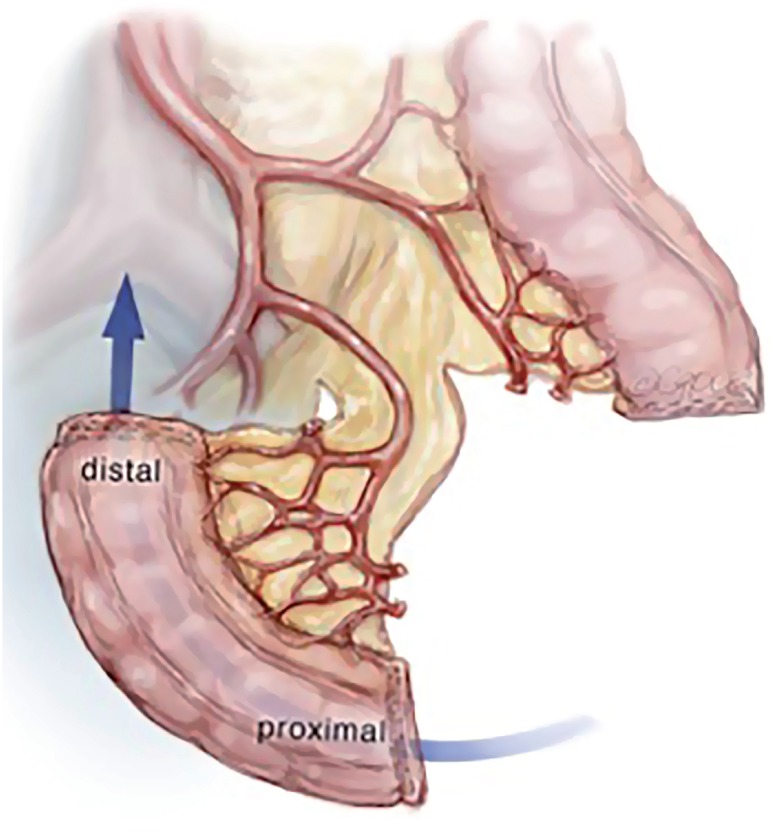
Mobilization of sigmoid conduit for urinary diversion.

**Figure 4:**
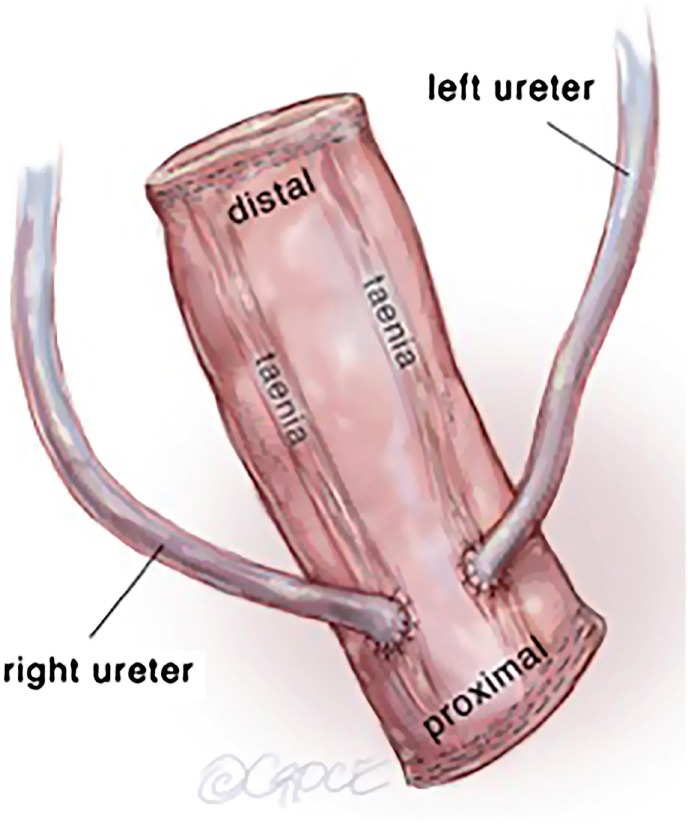
Uretero-sigmoid anastomosis.

**Figure 5:**
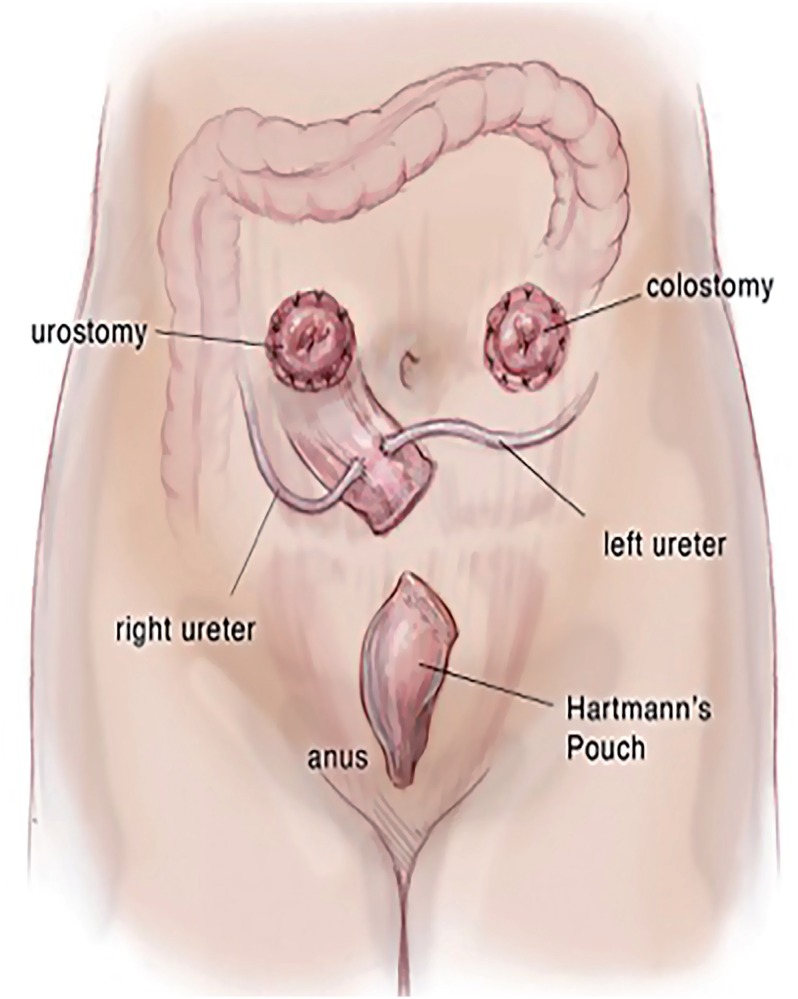
Final urinary and bowel diversion orientation.

In review of the surgical anatomy and blood supply of the large intestine, we pay close attention to the branches of the inferior mesenteric artery, the left colic artery to the descending colon and the superior hemorrhoidal artery to the sigmoid colon and rectum (Figure-1). The expertise of a general or colorectal surgeon to delineate the mesenteric blood supply and help decide where to make the mesenteric windows cannot be understated. The descending colon is mobilized from its retroperitoneal attachments, and a division site between the descending colon and sigmoid colon is chosen. The rectosigmoid junction is identified. The GIA stapling device is utilized to divide the colon at the junction of the descending and sigmoid colon and also at the rectosigmoid junction. The rectosigmoid mesentery is divided using an energy device or ties radially from the bowel, dividing the arc of the superior hemorrhoidal artery as it passes the rectosigmoid junction. The mesentery at the junction of the descending and sigmoid colons are divided as well up to the bifurcation of the inferior mesenteric artery, taking care to preserve the both branches of the bifurcation of the inferior mesenteric artery (Figure-2). If mesenteric length limits reach of the descending colon to the abdominal wall, the left colic artery can be divided at the bifurcation of the inferior mesenteric artery, such that the descending colon will draw its blood supply from the middle colic artery via the marginal artery. The rectal stump is left in place as a Hartman's Pouch for patients with benign disease, and the sigmoid is now free for mobilization to the level of the skin (Figure-3). The rectum is resected prior to beginning the diversion for those with malignancy. The descending colon with the GIA staple line will later be used to fashion the colostomy. The ends of both ureters are spatulated for 1-2cm. Ureteroenteric anastomosis is performed in non-refluxing, running fashion with either 4-0 Vicryl or 4-0 PDS (Figure-4). The ureters are anastomosed to the taenia of the sigmoid conduit in a proximal location. Urinary diversion stents are utilized. Because the sigmoid colon is utilized, the left ureter does not require tunneling under the sigmoid mesentery as would be done for an ileal conduit. The distal end of the sigmoid conduit is then brought out through the skin, preferably in the right lower quadrant, and the stoma is created in a standard Brooke fashion (6). If there is difficulty reaching the right lower quadrant due to body habitus, tight mesentery, or other anatomic restriction (e.g., baclofen pump on the right side of the abdomen in one of our cases), the urostomy can be seated in the left lower quadrant with the plan for the colostomy to be placed superiorly. The descending colon is then utilized for the colostomy in the left lower quadrant (Figure-5). The abdominal fascia is closed before opening the staple line and seating the colostomy.

### Case 1

SD is a 64 year-old gentleman with locally advanced rectal cancer who underwent neoadjuvant radiation therapy and was then lost to follow-up for planned surgical resection. He subsequently developed urinary retention with bilateral hydronephrosis in the setting of locally advanced disease without distant metastasis with prostatic and bladder base involvement of rectal cancer. After completing 10 cycles of FOLFOX chemotherapy, he consented to pelvic exenteration (cystoprostatectomy and abdominal-perineal rectal resection) with colostomy and concurrent sigmoid conduit urinary diversion. On September 2014, he underwent the above surgery with our described technique for urinary and bowel diversion. His postoperative course was complicated by acute blood loss anemia requiring blood transfusion and prolonged ileus, for which he received TPN and eventually tolerated a regular diet. He was discharged on POD#14.

### Case 2

DB is a 32 year-old T4 paraplegic male secondary to gunshot wound in 2008 with chronic decubitus ulcers, colitis and osteomyelitis of the hips. He had previously undergone multiple open wound debridements and myocutaneous flap coverage. He had neurogenic bladder managed with indwelling urethral catheter. He underwent urodynamics testing that showed a bladder capacity of only 44cc. Given his extensive decubitus ulcers, neurogenic bladder with small capacity, and ventral penile shaft erosion secondary to indwelling catheter, decision was made for cystectomy with urinary and intestinal diversion. He underwent the above surgery in January 2015 utilizing our described technique. We placed both the urostomy (inferior) and colostomy (superior) on the left side of the abdomen because of a subcutaneous baclofen pump located on the right side and a gastrostomy tube in the mid-upper abdomen. His postoperative course was remarkable for an ileus on POD#6 that resolved quickly when G tube was placed to gravity. He was advanced again to clear liquid diet on POD#8 and regular diet on POD#9. He was discharged on POD#9.

### Case 3

RR is a 52 year-old C5-C7 quadriplegic male with recurrent nephrolithiasis and neurogenic bladder and bowel. He experienced difficulty emptying his bladder despite a distant history of sphincterotomy and refused intermittent catheterization. During one of his ureteroscopic stone surgeries, he was found to have a flat bladder lesion on the floor of bladder that was biopsied and found to be invasive, moderately differentiated, squamous cell carcinoma. He was concurrently followed by colorectal surgery for hemorrhoids, rectal prolapse and atonic anal sphincter with chronic constipation from neurogenic bowel. Given his bladder disease, decision was made to address his colorectal conditions surgically to optimize his quality of life. In July 2015 he underwent radical cystoprostatectomy and urinary and bowel diversion with our described technique. Intraoperatively, his mesenteric vessels were notably robust. The proximal division was made just distal to the takeoff of the left colic artery from the inferior mesenteric artery. The sigmoid colon was then measured 20 centimeters and the peritoneum overlying the distal sigmoid was incised to increase length. The IMA and superior hemorrhoidal arteries were intact. Urostomy was seated on the right and colostomy on the left. His postoperative course was unremarkable. He was discharged to rehabilitation on POD#4 with no early or late complications.

### Case 4

DS is a 37 year-old non-smoking gentleman with cerebral palsy with multifocal, recurrent, high grade, non-muscle invasive urothelial carcinoma of the bladder which ultimately progressed to muscle invasive disease. At baseline, he voided independently but did exhibit signs of neurogenic bladder. His other comorbidities included sacral-decubitus ulcers and diarrhea-predominant irritable bowel syndrome (IBS) with fecal incontinence resulting in significant weight loss requiring gastrostomy tube placement. In August 2015, he underwent radical cystoprostatectomy with urinary and bowel diversion utilizing our described technique. Intraoperatively, there was notable difficulty with positioning due to flexion contractures. The sigmoid colon was able to be isolated for 15cm for a conduit. The colon was fortunately extremely redundant. The inferior mesenteric artery did need to be ligated for mobilization of the colonic segment. The mesentery was divided such that the superior hemorrhoidal artery supplied the sigmoid urinary conduit, and the left colic artery supplied the end colostomy. Post-operatively, he was slowly advanced to a regular diet on POD#5. His post-operative course was unremarkable and he was discharged on POD#8 with no early or late complications.

### Case 5

SA is a 63 year-old gentleman with locally advanced rectal carcinoma who underwent exploratory laparotomy and end sigmoid colostomy for large bowel obstruction. The primary tumor was seen on MRI to be locally invasive into the posterior prostate, seminal vesicles and perirectal lymph nodes. After completing chemoradiation, he was taken to the operating room for pelvic exenteration and urinary diversion. The plan was to leave the existing colostomy on the patient's left abdomen, and create an ileal conduit in the right hemi-abdomen, however, during the case the ileum appeared to have radiation related changes and the bowel appeared edematous and firm. Given that no significant segment of bowel appeared safe for removal and there was concern about well-healing bowel anastomosis, decision was made to use the existing colostomy as a urostomy. The colostomy was washed out thoroughly and the ureters were implanted near the taenia coli after the colon was divided 20cm from the ostomy. The remaining large bowel was then mobilized towards the right hemicolon and matured as a new transverse loop colostomy. The patient had an uneventful recovery with diet advanced to clears on POD#2, solids on POD#8 after return of bowel function. He was discharged on POD#10 with no complications.

Table-1 summarizes the five patients described above noting diagnosis, operative times, estimated blood loss (EBL), length of stay (LOS) and early (30 day) and late (90 day) complications by Clavien-Dindo classification.

**Table 1:** Case details for five cases with urinary diversions using colonic conduits.

Patient	Diagnosis	Operative times	EBL	LOS (days)	Early Complications ^7^ (30 days)	Late Complication ^7^ (90 days)
SD	Advanced rectal cancer involving prostate and bladder	10h 25m	1500 mL	14	Clavien II - acute blood loss with transfusion, TPN requirement for ileus	Clavien IIIB - Small bowel obstruction secondary to parastomal hernia requiring exploratory laparotomy
DB	T4 paraplegic with colitis, neurogenic bladder and decubitus ulcers	5h 39m	200 mL	9	Clavien IIIB - necrotizing right thigh fasciitis requiring debridement	None
RR	C5-7 quadriplegic with neurogenic bowel, rectal prolapse/ chronic constipation and invasive bladder squamous cell carcinoma	7h 0m	700 mL	4	None	None
DS	Cerebral palsy with high grade muscle-invasive urothelial carcinoma with irritable bowel syndrome	6h 6m	500 mL	8	None	None
SA	Locally invasive rectal adenocarcinoma into prostate and seminal vesicles	7h 1m	50 mL	10	None	None

## DISCUSSION

Though other groups have compared outcomes of simultaneous urinary and bowel diversion to staged surgery (7), the surgical technique has not been clearly described prior to this article. The surgical description with support from the five cases demonstrate the types of patients who may benefit from this operation.. The cases highlight minor modifications that can be pragmatic (e.g., placing both urostomy and colostomy on the same side of the abdomen is easily achieved, or using an existing conduit for urinary diversion, and re-siting the colon).

This short series emphasizes a team approach between urologic and general (or colorectal) surgeons. Too often in today's healthcare system is patient care fragmented among various specialists, and this can be most burdensome for patients that would benefit from multiple surgical procedures. For patients who would benefit from double diversion, efforts should be made to do concurrent surgery when possible for this higher-risk surgical population. Patient comorbid factors and malnutrition are strong incentives to minimize the number of surgical procedures. In addition, subsequent surgical procedures yield the added risk and complexity of adhesions and scarring.

For this surgical technique, the average OR times are variable but not outside the range for a standard cystectomy and urinary diversion. Interestingly, the four institutions involved in Romo's study found an increase in adverse events related to increase in operative time (7) for either simultaneous double diversion or staged diversions. In this small cohort, complications seemed similar to those seen after urinary diversion alone, but certainly more cases need to be completed to make any definitive conclusions. The major theoretical benefit is forgoing the bowel anastomosis. This simplifies the operation, may speed up recovery of bowel function, and virtually eliminates the risk of bowel leak in this malnourished group with a strong preponderance for slow fecal transit and constipation. By virtue of using the sigmoid colon, the left ureter is not tunneled under the mesentery to the right side. This can be very advantageous for obese patients where the traditionally tunneled left ureter is often placed on undesirable tension for the anastomosis. We observed no early or late ureteral strictures in our small series of 5 cases. In this patient population, adequate space to place two stomas is often at a premium. Body habitus, contractures, prior surgery, percutaneous drains (e.g., G-tube), and subcutaneous implants (e.g. baclofen pump) can limit the surface area required for both urostomy and colostomy. By utilizing the same mesentery for both diversions, placing both stomas on the same side of the abdomen is feasible, as described in one of our cases. This technique does largely rely on a mobile sigmoid colon and mesentery. The distal end of the sigmoid must be able to rotate significantly cephalad to reach the skin without tension. Other limitations, such as pelvic radiation affecting the sigmoid colon and its blood supply, can be potential factors that would preclude this operative technique. For advanced bladder cancer, radiation is less often utilized, but for gynecologic and colorectal malignancies this issue could arise more frequently.

In summary, we describe our surgical technique for concurrent urinary and bowel diversion utilizing the descending and sigmoid colon. The key steps of this operation are emphasized, in particular the management and appropriation of the blood supply to both the colostomy and urostomy. This technique avoids a bowel anastomosis, minimizes the amount of bowel taken out of continuity, allows for less tension on the left ureter, and provides more flexibility for where to place the urostomy. The authors believe this surgical technique is an excellent option for patients requiring both intestinal and urinary diversions.

## References

[1] 1. Hautmann RE. Urinary diversion: ileal conduit to neobladder. J Urol. 2003;169:834-42. 10.1097/01.ju.0000029010.97686.eb 12576795

[2] 2. Webster GD. Peterson AC. History of urinary diversion techniques. Urinary Diversion. Taylor and Francis, New York, 2005. 1-20.

[3] 3. Saber A. Urinary diversion: Historical aspect and patient's satisfaction. Urol. Nephrol. Open Access J 1.2014: 14-21.

[4] 4. Bricker EM. Bladder substitution after pelvic evisceration. Surg Clin North Am. 1950;30:1511-21. 10.1016/s0039-6109(16)33147-4 14782163

[5] 5. Warwick RT. Technique for the separate diversion of urine and faeces. Lancet. 1959;1:1021-2. 10.1016/s0140-6736(59)91260-7 13655662

[6] 6. Wein, Alan J., et al. “Use of intestinal segments in urinary diversion.” Campbell-Walsh urology 10th Edition (2012): 3:2411-449.

[7] 7. Barboglio Romo PG, Santiago-Lastra Y, Myers JB, Pathak P, Elliott SP, Cotter KJ, et al. Multi-institutional Outcomes for Simultaneous and Staged Urinary and Fecal Diversions in Patients Without Cancer. Urology. 2018;118:202-7. 10.1016/j.urology.2017.11.057 29366642

[8] 8. Dindo D, Demartines N, Clavien PA. Classification of surgical complications: a new proposal with evaluation in a cohort of 6336 patients and results of a survey. Ann Surg. 2004;240:205-13. 10.1097/01.sla.0000133083.54934.ae PMC1360123 15273542

